# The Role of NLRP3 Inflammasome Activation Pathway of Hepatic Macrophages in Liver Ischemia–Reperfusion Injury

**DOI:** 10.3389/fimmu.2022.905423

**Published:** 2022-06-10

**Authors:** Tong Wu, Cheng Zhang, Tianfeng Shao, Jianzhong Chen, Diyu Chen

**Affiliations:** ^1^ School of Medicine, Zhejiang University, Hangzhou, China; ^2^ Division of Hepatobiliary and Pancreatic Surgery, Department of Surgery, The First Affiliated Hospital, Zhejiang University School of Medicine, Hangzhou, China; ^3^ Department of General Practice, Shaoxing Yuecheng District Tashan Street Community Health Service Center, Shaoxing, China; ^4^ Institute of Immunology, School of Medicine, Zhejiang University, Hangzhou, China

**Keywords:** inflammasome, NLRP3, ischemia reperfusion injury, hepatic macrophage, liver transplantation

## Abstract

Ischemia-reperfusion injury (IRI) is considered an inherent component involved in liver transplantation, which induce early organ dysfunction and failure. And the accumulating evidences indicate that the activation of host innate immune system, especially hepatic macrophages, play a pivotal role in the progression of LIRI. Inflammasomes is a kind of intracellular multimolecular complexes that actively participate in the innate immune responses and proinflammatory signaling pathways. Among them, NLRP3 inflammasome is the best characterized and correspond to regulate caspase-1 activation and the secretion of proinflammatory cytokines in response to various pathogen-derived as well as danger-associated signals. Additionally, NLRP3 is highly expressed in hepatic macrophages, and the assembly of NLRP3 inflammasome could lead to LIRI, which makes it a promising therapeutic target. However, detailed mechanisms about NLRP3 inflammasome involving in the hepatic macrophages-related LIRI is rarely summarized. Here, we review the potential role of the NLRP3 inflammasome pathway of hepatic macrophages in LIRI, with highlights on currently available therapeutic options.

## Introduction

The innate immunity acts as the first line of defense that recognizes and eradicates pathogens in human. It is implemented in the presence of pathogen-associated molecular patterns (PAMPs) (such as bacteria, viruses and parasites) or damage-associated molecular patterns (DAMPs) through pattern recognition receptors (PRRs) (1–4). In the past decades, various kinds of PRRs (inflammasomes) were discovered in succession, such as Nod-like receptor protein 1 (NLRP1), NLRP2, NLRP3, absent in melanoma 2 (AIM2) and NLR family CARD domain containing 4 (NLRC4) (5, 6). As the most well studied inflammasome, NLRP3 has been confirmed to be a critical component that mediates caspase-1 activation and cleavage of gasdermin D (GSDMD) in response to microbial infection and specific endogenous danger-related stimuli (7–11). Activated GSDMD facilitate the formation of pore in the plasma membrane and trigger the pyroptotic cell death and enhance the secretion of inflammatory cytokines including IL-1β and IL-18 (12).

At present, liver transplantation (LT) is widely used as the most effective and definitive treatment for end-stage liver diseases, hepatic malignancies, acute fulminant hepatic failure, and metabolic disorders (13). During the LT surgery, the cell death in donor liver was exacerbated following the restoration of oxygen delivery. This special pathological disorder is concepted as liver ischemia reperfusion (I/R) injury (LIRI) (14). Due to the molecular characteristics, liver I/R could be categorized into two distinct stages. Followed by reopening of the spontaneous shunts, hepatocytes are deprived suddenly of oxygen and nutrient interruption. And this period is called ischemia stage. Then extended ischemia duration would lead to substantial parenchymal cell death. The other stage is reperfusion stage, in which the innate immunity and sterile inflammatory response are intensified. The macrophages in liver such as Kupffer cells (KCs) can be activated and play an important role in the context of LIRI (15). Recent studies have demonstrated that blocking the local inflammatory response in liver could effectively reduce LIRI. Thus, it is necessary to explore the potential strategies to prevent the innate immunity and inflammatory response activation during LIRI.

This review summarizes the recent advances in our understanding of the activation and regulation of the NLRP3 inflammasome activation, as well as its role in LIRI with highlights on the prevention of LIRI by inhibiting NLRP3 inflammasome aberrant activation.

## The NLRP3 Inflammasome

The NLRP3 inflammasome complex consists of an amino-terminal pyrin domain (PYD), a central nucleotide-binding and oligomerization domain (NOD), and a C-terminal leucine-rich repeat (LRR) domain (7, 16, 17). The pyrin domain of NLRP3 frequently referred to apoptosis-associated speck-like protein (ASC), which initiates the assembly of inflammasome.

The NLRP3 inflammasome can be activated by a wide range of stimuli that include Candida albicans, bacteria that produce pore-forming toxins such as Staphylococcus aureus and Listeria monocytogenes, viruses (such as influenza virus) (5, 18). Moreover, it is identified that host-derived molecules (including extracellular ATP, hyaluronan, fibrillar amyloid-β peptide, extracellular glucose, monosodium urate (MSU) crystals, and uric acid *etc.)* are involved in the NLRP3 inflammasome activation (18). On the basis of structural and chemical differences between these stimuli, it is suggested that NLPR3 senses common cellular events induced by its stimuli, but does not directly bind to it. However, there is still no strong evidence to support this hypothesis. Currently, a two-signal model has been proposed for NLRP3 inflammasome activation. The first signal is responsible for initiating the NLRP3 inflammasome, which including microbial components or endogenous cytokines. The second signal from pore-forming toxins, extracellular ATP, or particulate matter activates the NLRP3 inflammasome.

**Figure 1:**
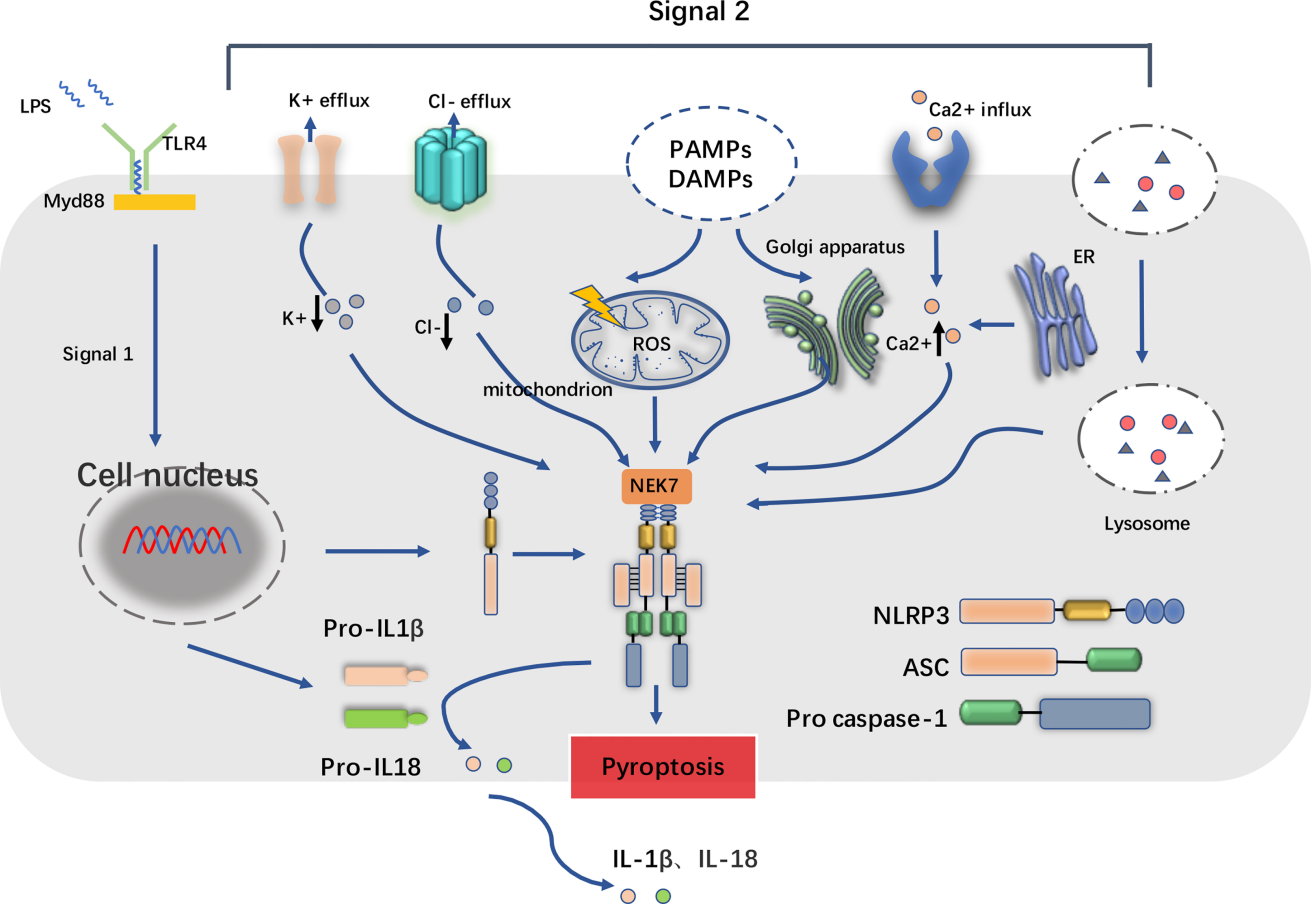
Basic structure of NLRP3 inflammasome and its two-step process of priming and activation. The NOD-like receptor family pyrin domain containing 3 (NLRP3) inflammasome complex consists of NLRP3 monomers, apoptosis-associated speck-like protein containing a CARD (ASC) and the pro casepase-1. When inflammatory stimuli such as lipopolysaccharide (LPS) sensed by Toll-like receptors (TLRs) (e.g., TLR4) and cytokine receptors, the downstream singling adaptors such as MyD88 cooperatively contribute to the priming process, which upregulate the transcription of NLRP3 and IL1β/IL18, accumulate cytoplasmic pro-IL1β/pro-IL18. In addition, NEK7, one of the serine/threonine kinases, is also required for the activation of NLRP3 inflammasome. In the second step, pathogen-associated molecular patterns (PAMPs) and damage-associated molecular patterns (DAMPs) trigger cellular events including reactive oxygen species (ROS) release, endoplasmic reticulum stress, mitochondrial dysfunction, lysosomal damage, and ionic flux such as K+ efflux, Cl- efflux and Ca2+ influx. These facilitate the cleavage of pro casepase-1 into active form casepase-1, and in turn the maturation and release of IL1β/IL18, consequently leading to the inflammatory cell death known as pyroptosis.

The excessive molecular and cellular signaling events have been clearly recognized as the major consequences of NLRP3 stimuli, compromising reactive oxygen species (ROS), mitochondrial dysfunction, lysosomal damage, and ionic flux (10, 19). Proposed as the common signal for NLRP3 inflammasome activation, it is observed that the level of ROS increased after the NLRP3 stimuli treatment. Lysosomal NADPH oxidase is deemed to be the origin of ROS generation, and the accumulation process of ROS may be caused by respiratory function, which is closely related to the activation of inflammasome. Furthermore, it is illustrated that mitochondrial ROS (mtROS) is also a kind of production in dysfunctional mitochondria (20). As an essential element, mtROS can be continuously increased with the release of LPS and ATP and mitochondrial DNA (mtDNA) into the cytoplasm. This process is important for the mtROS-dependent manner of NLRP3 inflammasome activation. Furthermore, some kinds of particulate matter (such as MSU, alum, silica, asbestos, amyloid-β, cholesterol crystals, and calcium crystals) induces NLRP3 inflammasome activation in macrophages. Moreover, lysosomal damage after phagocytosis results in the leakage of lysosomal contents into the cytosol. However, the mechanism involves in lysosomal disruption to NLRP3 inflammasome activation remains unclear. Lysosomal acidification, leakage of active lysosomal enzymes such as Cathepsins B, L, C, S, and X may account for NLRP3 inflammasome activation. The ionic flux events, such as K^+^ efflux, Ca^2+^ mobilization, Cl^−^ efflux, and Na^+^ influx, could all be accelerated by NLRP3 stimuli, which are implicated in maintaining the NLRP3 inflammasome activity (**Figure 1**).

## Hepatic Macrophage Participating in Liri

Hepatic macrophages play an important role in the pathogenesis of LIRI and have been proposed as the primary cells for NLRP3 activation (15). Recent studies have revealed that hepatic macrophages are a heterogeneous population of innate immune cells. According to the molecular characteristics, hepatic macrophages can be categorized into liver-resident (KCs and liver capsular macrophages (LCMs)) and non-resident (monocyte-derived and peritoneal macrophages) cells (15).

KCs account for 20%∼35% of all non-parenchymal cells in the liver and 80%∼90% of tissue macrophages presented in the body. KCs are marked as CD45^+^F4/80^high^CD11b^low^CLEC4F^+^ cells in mice (21). Analyzing the single-cell RNA-sequencing data, the researchers identified two distinct subtypes of KCs in liver: the immunoregulatory KCs and the pro-inflammatory KCs (22).

Act as the microenvironment sensor, KCs resides at the luminal side of the hepatic sinusoidal endothelium. They are essential for maintaining local homeostasis, such as clearance of pathogens of systemic and gut origin, and regulation of iron metabolism. KCs play a vital role in the liver homeostasis by utilizing followed functions: (i) clearance of metabolic waste and cellular debris (22–24); (ii) preservation of iron homeostasis *via* engulf of red blood cells and the subsequent recycling of iron (25–28); (iii) regulation of cholesterol metabolism through the production of cholesteryl ester transfer proteins (29); (iv) mediation of antimicrobial defense (30, 31) and (v) promotion of immunological tolerance.

LCMs are newly identified murine liver-residents of the CD11b^+^F4/80^+^CX3CR1^+^MHC II^+^ phenotype. LCMs do not express classic Kupffer cell (TIM4 and CLEC4F) or monocyte-derived macrophage (Ly6C) markers and form a contiguous cellular network in the hepatic capsule (23). LCMs sense peritoneal bacteria and promote recruitment of neutrophil into the capsule. When LCMs populated in the hepatic sinusoids, they are responsible for monitoring the expand of intra-peritoneal bacteria. In order to avoid the exhaustion of LCMs, blood monocytes could supply and mature LCMs in the steady-state. Despite the growing recognition of the importance of LCMs in hepatic pathogen defense, whether LCMs play a role in LIRI-induced sterile inflammation in the liver remains unclear and needs to be further addressed.

Monocyte-derived macrophages (MoMϕs) are differentiated from bone marrow (BM) CX3CR1^+^CD117^+^Lin^−^ progenitor cells-derived circulating monocytes. In mice, MoMϕs are CD11b^+^, F4/80intermediate (int), Ly6C^+^ and CSF1R^+^ while KCs are CD11b^low^, F4/80^high^ and Clec4F^+^ (21, 24). Hepatic MoMϕs could be classified into two main subpopulations on the basis of Ly6C expression: Ly6C high and Ly6C low MoMϕs in mouse models of liver diseases,

Based on the recent single-cell RNA-sequencing results, it is illustrated that CD68^+^MARCO^+^ KCs, CD68^+^MARCO^−^ macrophages, and CD14^+^ monocytes are specifically enriched in the liver microenvironment (26). Through the further integrative analysis, it is also found that CD68^+^MARCO^+^ KCs could be recognized by immune tolerance (e.g., VSIG4) and inflammation inhibiting (e.g., CD163 and HMOX1) related genes. CD68^+^MARCO^−^ macrophages as recruited proinflammatory macrophages have a similar transcriptional profile (e.g., C1QC, IL-18, S100A8/9). However, peripheral CD14^+^ monocytes show significantly proinflammatory responses than both CD68^+^ MARCO^−^ macrophages and hepatic CD14^+^ monocytes.

In the steady environment, KCs self-renew *via* homeostatic repopulation (27). Under cellular stress, the self-renewal of KCs would be hampered, which results in the suppression of their homeostatic repopulation. Sterile inflammation (such as LIRI) decrease KC numbers, which would be replaced by bone marrow-derived monocytes replacing in a mouse LIRI model (28). Notably, infiltrating Ly6C^high^-CCR2^high^-CX3CR1^low^ monocyte-derived macrophages (MoMFs) show great plasticity maintaining phenotype and functions. In mouse models after depletion of Kupffer cell by diphtheria toxin receptor (DTR), MoMFs accumulate the hepatic macrophage differentiated towards functional and self-renewing KCs (21).

**Figure 2:**
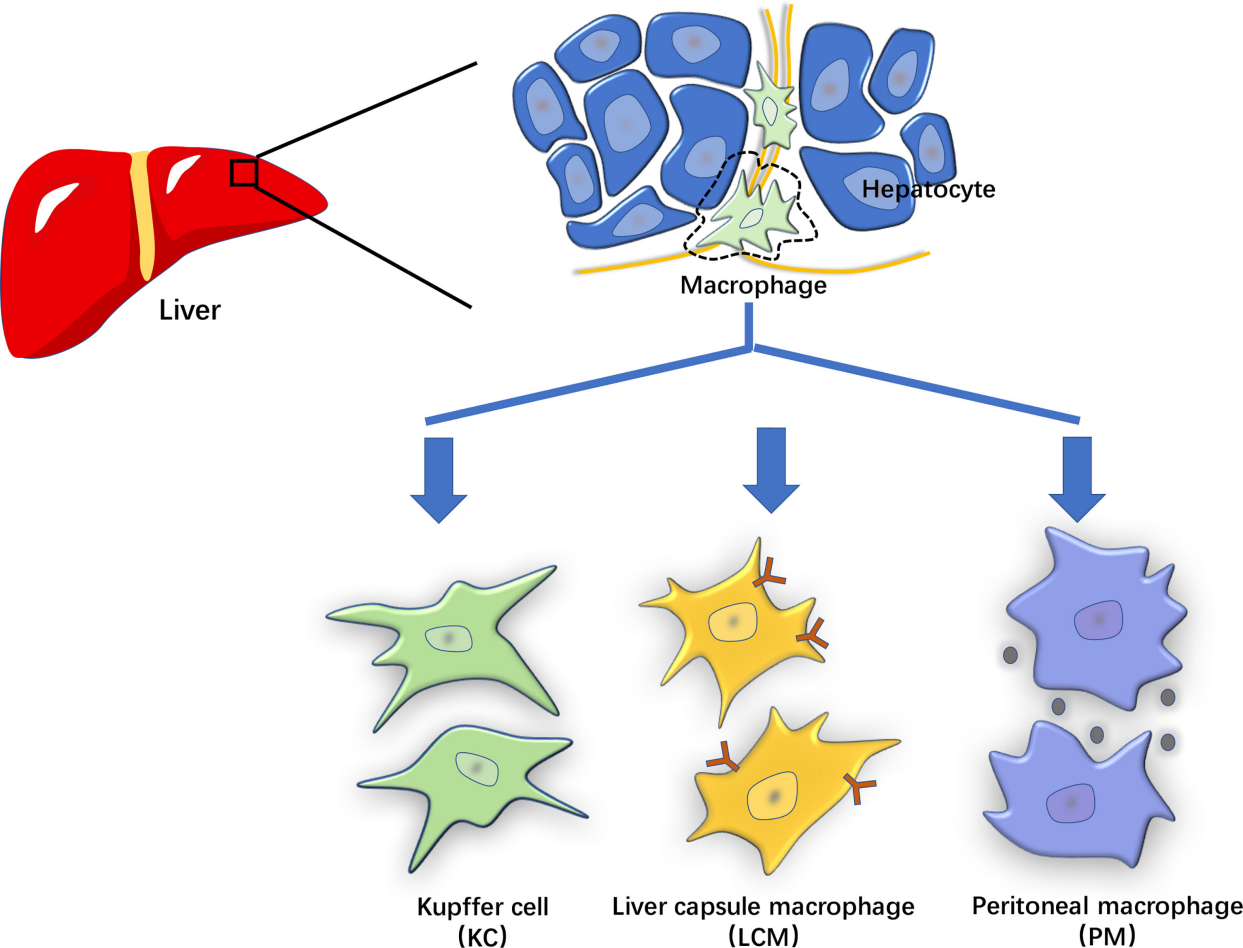
The characterization of hepatic macrophages. The hepatic macrophages based on their origin and molecular features can be classified into liver-resident cells such as Kupffer cells (KCs) and liver capsular macrophages (LCMs), and non-resident cells such as monocyte-derived and peritoneal macrophages (PMs).

Since their depletion promotes liver inflammation, KCs undergoing cell death in response to LIRI may affect homeostasis and activate inflammatory macrophage in mice (28). Being in contact with DAMPs produced by dead or moribund LIRI-stressed cells, KCs are activated through sensing early organ damage *via* PRRs (**Figure 2**). Then these activated KCs secrete cytokines and chemokines that recruit circulating monocytes, neutrophils and T cells to promote liver injury (29). Liver sterile inflammation recruits new populations of extrahepatic macrophages (emergency repopulation) such as MoMFs and peritoneal macrophages. These monocytes consist of a bone marrow-derived pro-inflammatory Ly6C^high^CCR2^high^CX3CR1^low^ subset and an anti-inflammatory Ly6C^low^CCR2^low^CX3CR1^high^ subset in mice (27). Human monocytes are similarly defined as CX3CR1^low^CD14^+^CD11b^high^CD11c^+^CD62L^+-^CD16^low^ or CX3CR1^high^CD14^low^CD16^+^CD11b^+^CD11c^high^ subsets. In summary, the above hepatic macrophages play a proinflammatory effect after sensing DAMPs and mediate pathogenesis in LIRI.

## The NLRP3 ACTIVATION of Liver Macrophages in Liri

Constant monitoring for infection or non-infectious threats to tissue integrity is one of the major immune functions of liver-derived KCs (30). During hepatic injury, KCs can promote inflammasome formation while recognizing DAMPs or PAMPs that bind to PRRs such as NLRs. In the early stage of LIRI, ischemic injury leads to hypoxia in hepatocytes, which further causes pH changes and ATP depletion. These changes can increase the liver’s dependence on glycogen for energy production. At the same time, these events mentioned above promote the release of ROS, increase the intracellular calcium concentration, and exacerbate organelle damage, ultimately leading to cell damage or death.

Reperfusion after liver transplantation can enhance the inflammatory cascade, thereby aggravating liver injury. Furthermore, the destruction and death of hepatocytes and sinusoidal endothelial cells can trigger the release of DAMPs such as ATP and HMGB1. These DAMPs potentially activate hepatic macrophages such as KCs to awaken innate inflammatory responses, which may assemble the NLRP3 inflammasome and activate pyroptosis-regulated signaling pathways (31). These macrophages are critical in the mediation of LIRI, not only for recognizing damage-associated molecules to initiate inflammation and recruit immune cells, but also to help end inflammation and repair tissue damage.

The potential role of NLRP3 inflammasome activation pathway in LIRI has been investigated both *in vitro* and *in vivo* (32–34). Through the experiments, it was confirmed that the activation of NLRP3 inflammasome play a vital part in followed inflammatory response (33, 35). But utilizing the NLRP3 inflammasome inhibitors may mitigate hepatic inflammation through different signaling pathway.

Since both NLRP3 and caspase-1 knockout attenuated the inflammatory response in the LIRI mouse model, and these two genes are upregulated during injury. Thus, it is thought that NLRP3 activation is critical for LIRI progression (32, 33). The knockdown of NLRP3 in model mice decreased the serum alanine aminotransferase levels, lowered the secretion of proinflammatory cytokines (such as IL-1β, IL-18, TNF-α, and IL-6) and inhibited the release of HMGB1. Overall, NLRP3 knockdown decreased the infiltration of inflammatory cells and protected the liver from I/R injury. Furthermore, depletion of myeloid cell-specific GSDMD suppressed warm LIRI, suggesting that macrophage and neutrophil pyroptosis have a driving role during hepatic ischemic stress (36). In the further study, some studies discovered that NLRP3 is highly expressed in macrophages and monocytes while downregulated in hepatocytes and stellate cells, which also indicating that macrophages are the main effectors (37–39). Liver I/R stimuli upregulates NLRP3, but not ASC that containing a caspase recruitment domain. This suggests a feed-back pathway of NLRP3 inflammasome activation exists in LIRI, and it might be exploited for therapeutic purpose (35).

It is reported that aged liver receivers are sensitive to the liver I/R injury. Using integrative analysis, Zhong et al. showed that the STING-NLRP3 pathway is responsible for the proinflammatory response of aged macrophages in elderly patients (40). Additionally, aggravated liver I/R injury was found in db/db mice with increased ROS expression. N-Acetyl-L-cysteine (NAC) treatment significantly inhibited hepatocyte NLRP3 inflammasome activation and pyroptosis in db/db mice after I/R, suggesting that ROS plays an important role in mediating hepatocyte pyroptosis in the diabetic setting (41).

Studies have shown that both exosomes and pyroptosis play a role in LIRI and are essential in neuronal death. In the LIRI rat model, the NLRP3 inflammasome is activated and caspase-1-dependent pyroptosis occurs in the hippocampus and cortex. Serum-derived exosomes from LIRI-challenged rats not only penetrated the blood-brain barrier (BBB) but also caused neuronal cell pyroptosis. Furthermore, in the exosome challenge group, ROS and malondialdehyde (MDA) production were induced, while the NLRP3 inhibitor (MCC950) attenuated LIRI-mediated pyroptosis of hippocampal and cortical neurons (42).

Robust activation of the NLRP3 inflammasome was demonstrated in KCs during LIRI (33). Huang et al. showed that during liver I/R, endogenous extracellular histones activate the NLRP3 inflammasome in KCs through TLR9-dependent production of ROS. Activation of the NLRP3 inflammasome can also regulate neutrophils and inflammatory monocytes infiltrating the liver after I/R. However, loss of NLRP3 provides a stable innate immune environment. Also, the numbers of DCs, neutrophils and inflammatory monocytes remained unchanged compared to liver I/R. Sham KO mice. These data suggest that depletion of the NLRP3 inflammasome downregulates the innate immune response by reducing the influx of innate immune cells in the ischemic lobe after liver I/R (33). Inhibition of the NLRP3 inflammasome also attenuates I/R-mediated hepatocyte injury and prevents several pro-inflammatory cytokines such as IL-1β, IL-18, HMGB1 and IL-6 by preventing the stimulation of caspase-1, and the release of NF-κB pathway. Inoue et al. found that NLRP3(-/-) neutrophils reduced the concentration of keratinocyte-derived chemokine-induced intracellular calcium, ROS activation, and actin assembly formation, resulting in impaired migratory activity. NLRP3 can regulate chemokine-mediated functions and neutrophil recruitment, leading to liver I/R injury independent of the inflammasome. These discoveries reveal a novel role for NLRP3 in the pathophysiology of LIRI (35).

Activation of NLRP3 in LIRI is regulated by various signaling pathway. The heat shock factor 1 (HSF1)-β-catenin axis mediates the activation of NLRP3 by regulating the X-box binding protein 1 (XBP1) signaling axis. HSF1 activation promoted β-catenin expression, which in turn inhibited XBP1, resulting in NLRP3 inactivation and LIRI mitigation (43). Recently, dexmedetomidine was reported to show therapeutic potential by repressing the activation of NLRP3 inflammasome and alleviating LIRI *via* the miR- 494/JUND/PI3K/AKT/Nrf2 axis (44).

Thioredoxin-interacting protein(TXNIP) is an oxidative sensor that in homeostasisand is released after ROS stimulation (45)., TXNIP interacts with NLRP3 to promote its activation. Hypothermic oxygenated perfusion (HOPE) may modulate the TXNIP/NLRP3 inflammasome pathway from which liver injuries were reduced and liver function improved (46).

Autophagy, characterized by conserved autophagy, contributes to the degradation of intracellular components such as dysfunctional organelles, macromolecular complexes, long-lived cytoplasmic proteins, and foreign bodies. Recent studies have shown that autophagy negatively regulates the activation of the NLRP3 inflammasome during LIRI. Xue et al. reported that the antioxidant lycopene, elevated autophagosomes and increased protein levels of LC3B in KCs, and the autophagy inhibitor 3-methyladenine blocked the inhibitory effect of lycopene on the NLRP3 inflammasome in KCs (47). It is further demonstrated that lycopene promoted Nrf2/heme oxygenase 1 (HO-1) pathway activation and suppressed the NLRP3 inflammasome activation *via* enhancing KC autophagy (47). The expression of V-ATPase D2 subunit (ATP6V0D2) in liver macrophages was upregulated after LIRI which can promote the formation of autophagolysosomes to increase autophagy flux to limit the activation of liver inflammation (48). Eva-1 homologous gene A (EVA1A) is a kind of lysosomal and endoplasmic reticulum-related protein that has been found to be involved in regulating autophagy and apoptosis (49). Knockdown of EVA1A in KCs inhibits the formation of autophagosomes through inhibiting formation of ATG5/ATG12 complex in I/R process. suppressed combined action with Atg16L1. Knockdown of transient receptor potential melastatin 2 (TRPM2) also prevents LIRI by inhibiting autophagy activation and NLRP3 inflammasome pathway, which is *via* the exogenous upregulation of LC3-II (50).

**Figure 3:**
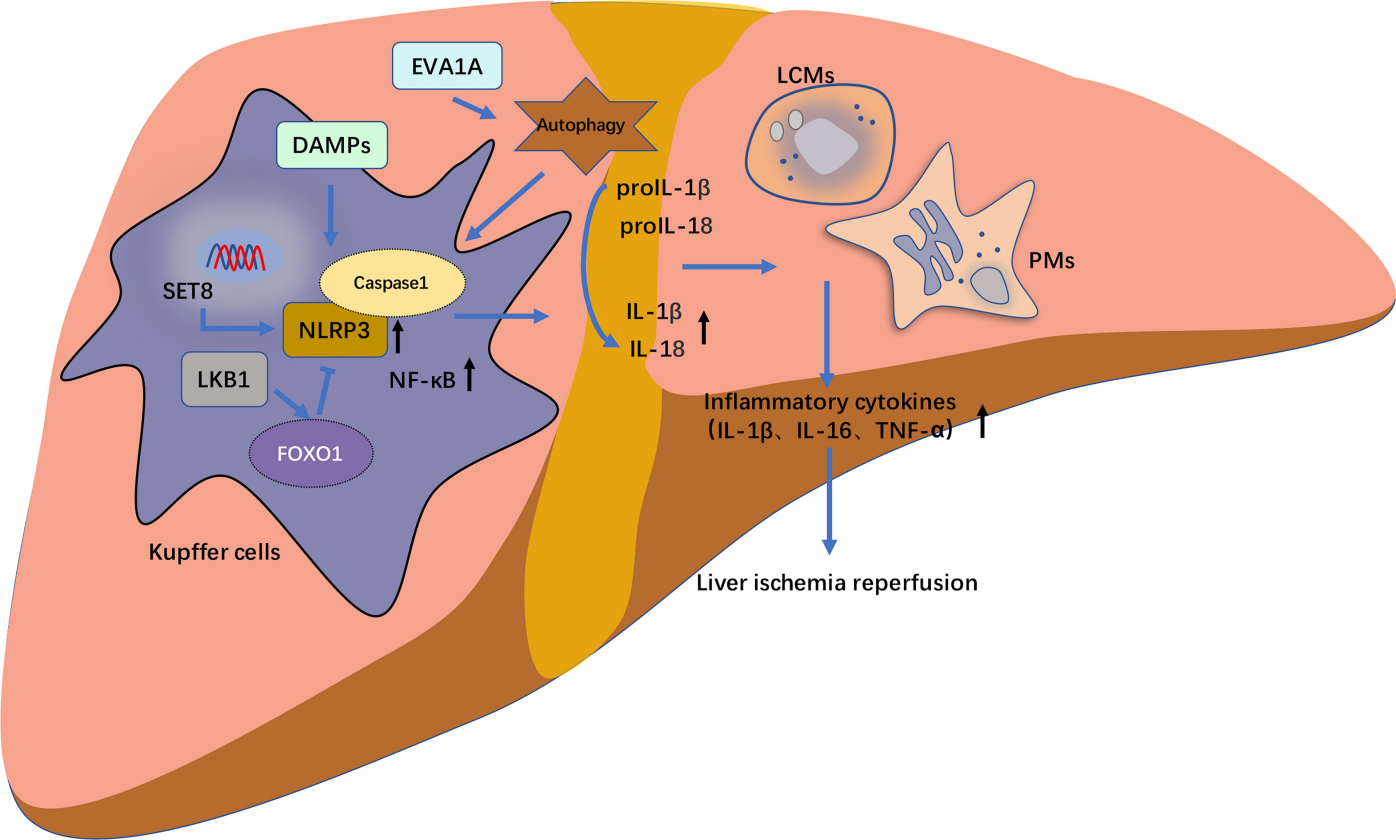
The schematic diagram of NLRP3 inflammasome activation pathway of hepatic macrophages in liver ischemia–reperfusion injury (LIRI). The release of caspase-1 and following cell death of pyroptosis mediated by NLRP3 inflammasome activation pathway frequently occurs in hepatic macrophages including Kupffer cells (KCs), liver capsular macrophages (LCMs), monocyte-derived and peritoneal macrophages (PMs). These hepatic macrophages regulate on another and modulate the progression of LIRI-related NLRP3 inflammasome activation through the release of inflammatory cytokines such as IL1β, IL18 and TNF-α. In RAW 264.7 cells (mice macrophages), SET domain-containing protein 8 (SET8) negatively regulate LIRI inflammatory responses and ameliorate liver injury through the inhibition of MARK4/NLRP3 inflammasome activation pathway. Eva-1 homologous gene A (EVA1A) is upregulated in inflammatory responses of LIRI in Kupffer cells (KCs), in which its overexpression induces more formation of autophagosomes. Whereas EVA1A silencing can promote ASC activation and increase the cleavage of caspase 1 and IL1β by activating autophagy. Liver kinase B1 (LKB1) alleviates LIRI by inhibiting the activation of NLRP3 inflammasome and NF-κB. Additionally, this inhibitory effect could be mediated through the forkhead box protein O1 (FOXO1).

Abundant regulators inhibit NLRP3 inflammasomes activation to alleviate LIRI by regulating different pathways (51–62). Zarpou et al. found that Silibinin ameliorated inflammatory liver tissue injuries, including neutrophil and macrophage infiltration, hepatocyte degeneration, cytoplasmic vacuolation, vascular endothelial damages, and sinusoid dilation observed in the I/R group (51). Bruton’s tyrosine kinase (BTK) is mainly expressed on KCs and sinusoidal endothelial cells, and BTK inhibitor ibrutinib effectively attenuates liver I/R injury by suppressing activation of the NLRP3 inflammasome in KCs (52). T3 and fisetin suppressed I/R liver injury-dependent AMPK pathway (63, 64). While the 12-hours fasting exerted beneficial effects on the prevention of LIRI by increasing serum β-hydroxybutyric acid (BHB), thus up regulated forkhead box transcription factor O1 (FOXO1) and HO-1, and by reducing the inflammatory responses and apoptotic cell death *via* the down-regulation of NF-κB and NLRP3 inflammasome (65). SET domain-containing protein 8 (SET8) negatively regulates liver I/R-mediated inflammatory response and ameliorates LIRI by suppressing microtubule affinity-regulating kinase 4 (MARK4)/NLRP3 inflammasome pathway (66). Docosahexaenoic acid (DHA) ameliorated I/R-induced injury by inhibiting pyroptosis of hepatocytes induced in liver I/R injury *in vivo* and *in vitro* through the PI3K/Akt pathway, providing a potential therapeutic option to prevent LIRI (67). The histone deacetylase Sirtuin-1 (SIRT1) inhibits the downstream XBP1/NLRP3 inflammatory pathway by activating miR-182, thus alleviating LIRI in mice (**Figure 3**) (68).

Isoflurane preconditioning significantly relieved liver IRI in mice and LPS-induced inflammation in liver macrophages by reduced intracellular Ca^2+^ levels, NF-κB translocation, and NLRP3 inflammasome activation in LPS-induced macrophages (69, 70). Iisoflurane pretreatment also inhibited caspase-11 expression and noncanonical pyroptosis-related production of cytokines (IL-1beta and IL-18). These findings suggest that isoflurane could be a pharmacological agent for liver IRI prevention and thus deserves more attention and further investigation (71).

## Conclusion

Recent advances continue to improve our understanding of the mechanisms involved in activation of NLRP3 inflammasome in LIRI. The KCs play a main part in the process. However, the pathological sequences of other macrophages such as recently identified LCM and other macrophages need to be further investigated. The advance in the subject also will improve the development of clinical strategies against LIRI and prevention of liver transplantation failure.

## Author Contributions

TW: data analysis and writing. TS: statistics and data collection. CZ: statistics and program guidance. JC and DC: program guidance and supervision. All authors contributed to the article and approved the submitted version.

## Funding

This study was supported by grants issued by the National Natural Science Foundation of China (82000618), Medical and Health Science and Technology Project of Zhejiang Province (2019KY290, 2021KY024).

## Conflict of Interest

The authors declare that the research was conducted in the absence of any commercial or financial relationships that could be construed as a potential conflict of interest.

## Publisher’s Note

All claims expressed in this article are solely those of the authors and do not necessarily represent those of their affiliated organizations, or those of the publisher, the editors and the reviewers. Any product that may be evaluated in this article, or claim that may be made by its manufacturer, is not guaranteed or endorsed by the publisher.

## References

[1] Gong T Liu L Jiang W Zhou R. DAMP-Sensing Receptors in Sterile Inflammation and Inflammatory Diseases. Nat Rev Immunol (2020) 20:95–112. doi: 10.1038/s41577-019-0215-7 31558839

[2] Zindel J Kubes P. DAMPs, PAMPs, and LAMPs in Immunity and Sterile Inflammation. Annu Rev Pathol (2020) 15:493–518. doi: 10.1146/annurev-pathmechdis-012419-032847 31675482

[3] Brubaker SW Bonham KS Zanoni I Kagan JC. Innate Immune Pattern Recognition: A Cell Biological Perspective. Annu Rev Immunol (2015) 33:257–90. doi: 10.1146/annurev-immunol-032414-112240 PMC5146691 25581309

[4] Janeway CA Jr. Medzhitov R. Innate Immune Recognition. Annu Rev Immunol (2002) 20:197–216. doi: 10.1146/annurev.immunol.20.083001.084359 11861602

[5] Martinon F Mayor A Tschopp J. The Inflammasomes: Guardians of the Body. Annu Rev Immunol (2009) 27:229–65. doi: 10.1146/annurev.immunol.021908.132715 19302040

[6] Ross C Chan AH von Pein JB Maddugoda MP Boucher D Schroder K. Inflammatory Caspases: Toward a Unified Model for Caspase Activation by Inflammasomes. Annu Rev Immunol (2022) 40:249–69. doi: 10.1146/annurev-immunol-101220-030653 35080918

[7] Huang Y Xu W Zhou R. NLRP3 Inflammasome Activation and Cell Death. Cell Mol Immunol (2021) 18:2114–27. doi: 10.1038/s41423-021-00740-6 PMC8429580 34321623

[8] Xu T Du Y Fang XB Chen H Zhou DD Wang Y. New Insights Into Nod-Like Receptors (NLRs) in Liver Diseases. Int J Physiol Pathophysiol Pharmacol (2018) 10:1–16. 29593846 PMC5871625

[9] Moretti J Blander JM. Increasing Complexity of NLRP3 Inflammasome Regulation. J Leukoc Biol (2021) 109:561–71. doi: 10.1002/JLB.3MR0520-104RR PMC8985609 32531835

[10] Swanson KV Deng M Ting JP. The NLRP3 Inflammasome: Molecular Activation and Regulation to Therapeutics. Nat Rev Immunol (2019) 19:477–89. doi: 10.1038/s41577-019-0165-0 PMC7807242 31036962

[11] Maiorino L Dassler-Plenker J Sun L Egeblad M. Innate Immunity and Cancer Pathophysiology. Annu Rev Pathol (2022) 17:425–57. doi: 10.1146/annurev-pathmechdis-032221-115501 PMC9012188 34788549

[12] Liu X Zhang Z Ruan J Pan Y Magupalli VG Wu H. Inflammasome-Activated Gasdermin D Causes Pyroptosis by Forming Membrane Pores. Nature (2016) 535:153–8. doi: 10.1038/nature18629 PMC5539988 27383986

[13] Kumar R Anand U Priyadarshi RN. Liver Transplantation in Acute Liver Failure: Dilemmas and Challenges. World J Transplant (2021) 11:187–202. doi: 10.5500/wjt.v11.i6.187 34164294 PMC8218344

[14] Papadopoulos D Siempis T Theodorakou E Tsoulfas G. Hepatic Ischemia and Reperfusion Injury and Trauma: Current Concepts. Arch Trauma Res (2013) 2:63–70. doi: 10.5812/atr.12501 24396796 PMC3876547

[15] Hirao H Nakamura K Kupiec-Weglinski JW. Liver Ischaemia-Reperfusion Injury: A New Understanding of the Role of Innate Immunity. Nat Rev Gastroenterol Hepatol (2021) 19:239–56. doi: 10.1038/s41575-021-00549-8 34837066

[16] Ohto U Kamitsukasa Y Ishida H Zhang Z Murakami K Hirama C. Structural Basis for the Oligomerization-Mediated Regulation of NLRP3 Inflammasome Activation. Proc Natl Acad Sci U.S.A. (2022) 119:e2121353119. doi: 10.1073/pnas.2121353119 35254907 PMC8931350

[17] Rahman T Nagar A Duffy EB Okuda K Silverman N Harton JA. NLRP3 Sensing of Diverse Inflammatory Stimuli Requires Distinct Structural Features. Front Immunol (2020) 11:1828. doi: 10.3389/fimmu.2020.01828 32983094 PMC7479093

[18] Schroder K Tschopp J. The Inflammasomes. Cell (2010) 140:821–32. doi: 10.1016/j.cell.2010.01.040 20303873

[19] Sharma BR Kanneganti TD. NLRP3 Inflammasome in Cancer and Metabolic Diseases. Nat Immunol (2021) 22:550–9. doi: 10.1038/s41590-021-00886-5 PMC8132572 33707781

[20] Zhou R Yazdi AS Menu P Tschopp J. A Role for Mitochondria in NLRP3 Inflammasome Activation. Nature (2011) 469:221–5. doi: 10.1038/nature09663 21124315

[21] Scott CL Zheng F De Baetselier P Martens L Saeys Y De Prijck S. Bone Marrow-Derived Monocytes Give Rise to Self-Renewing and Fully Differentiated Kupffer Cells. Nat Commun (2016) 7:10321. doi: 10.1038/ncomms10321 26813785 PMC4737801

[22] MacParland SA Liu JC Ma XZ Innes BT Bartczak AM Gage BK. Single Cell RNA Sequencing of Human Liver Reveals Distinct Intrahepatic Macrophage Populations. Nat Commun (2018) 9:4383. doi: 10.1038/s41467-018-06318-7 30348985 PMC6197289

[23] Sierro F Evrard M Rizzetto S Melino M Mitchell AJ Florido M. A Liver Capsular Network of Monocyte-Derived Macrophages Restricts Hepatic Dissemination of Intraperitoneal Bacteria by Neutrophil Recruitment. Immunity (2017) 47:374–388.e376. doi: 10.1016/j.immuni.2017.07.018 28813662

[24] Krenkel O Tacke F. Liver Macrophages in Tissue Homeostasis and Disease. Nat Rev Immunol (2017) 17:306–21. doi: 10.1038/nri.2017.11 28317925

[25] Takenaka E Van Vo A Yamashita-Kanemaru Y Shibuya A Shibuya K. Selective DNAM-1 Expression on Small Peritoneal Macrophages Contributes to CD4(+) T Cell Costimulation. Sci Rep (2018) 8:15180. doi: 10.1038/s41598-018-33437-4 30315271 PMC6185969

[26] Zhao J Zhang S Liu Y He X Qu M Xu G. Single-Cell RNA Sequencing Reveals the Heterogeneity of Liver-Resident Immune Cells in Human. Cell Discovery (2020) 6:22. doi: 10.1038/s41421-020-0157-z 32351704 PMC7186229

[27] McDonald B Kubes P. Innate Immune Cell Trafficking and Function During Sterile Inflammation of the Liver. Gastroenterology (2016) 151:1087–95. doi: 10.1053/j.gastro.2016.09.048 27725145

[28] Yue S Zhou H Wang X Busuttil RW Kupiec-Weglinski JW Zhai Y. Prolonged Ischemia Triggers Necrotic Depletion of Tissue-Resident Macrophages To Facilitate Inflammatory Immune Activation in Liver Ischemia Reperfusion Injury. J Immunol (2017) 198:3588–95. doi: 10.4049/jimmunol.1601428 PMC5419828 28289160

[29] Li P He K Li J Liu Z Gong J. The Role of Kupffer Cells in Hepatic Diseases. Mol Immunol (2017) 85:222–9. doi: 10.1016/j.molimm.2017.02.018 28314211

[30] Zannetti C Roblot G Charrier E Ainouze M Tout I Briat F. Characterization of the Inflammasome in Human Kupffer Cells in Response to Synthetic Agonists and Pathogens. J Immunol (2016) 197:356–67. doi: 10.4049/jimmunol.1502301 27226092

[31] Du Y Zhong F Cheng H Li T Chen Y Tan P. The Dietary Supplement Gamma-Oryzanol Attenuates Hepatic Ischemia Reperfusion Injury *via* Inhibiting Endoplasmic Reticulum Stress and HMGB1/NLRP3 Inflammasome. Oxid Med Cell Longev (2021) 2021:4628050. doi: 10.1155/2021/4628050 34512864 PMC8433023

[32] Zhu P Duan L Chen J Xiong A Xu Q Zhang H. Gene Silencing of NALP3 Protects Against Liver Ischemia-Reperfusion Injury in Mice. Hum Gene Ther (2011) 22:853–64. doi: 10.1089/hum.2010.145 21128730

[33] Huang H Chen HW Evankovich J Yan W Rosborough BR Nace GW. Histones Activate the NLRP3 Inflammasome in Kupffer Cells During Sterile Inflammatory Liver Injury. J Immunol (2013) 191:2665–79. doi: 10.4049/jimmunol.1202733 PMC3777242 23904166

[34] Kim HY Kim SJ Lee SM. Activation of NLRP3 and AIM2 Inflammasomes in Kupffer Cells in Hepatic Ischemia/Reperfusion. FEBS J (2015) 282:259–70. doi: 10.1111/febs.13123 25327779

[35] Inoue Y Shirasuna K Kimura H Usui F Kawashima A Karasawa T. NLRP3 Regulates Neutrophil Functions and Contributes to Hepatic Ischemia-Reperfusion Injury Independently of Inflammasomes. J Immunol (2014) 192:4342–51. doi: 10.4049/jimmunol.1302039 24696236

[36] Li J Zhao J Xu M Li M Wang B Qu X. Blocking GSDMD Processing in Innate Immune Cells But Not in Hepatocytes Protects Hepatic Ischemia-Reperfusion Injury. Cell Death Dis (2020) 11:244. doi: 10.1038/s41419-020-2437-9 32303674 PMC7165177

[37] Csak T Ganz M Pespisa J Kodys K Dolganiuc A Szabo G. Fatty Acid and Endotoxin Activate Inflammasomes in Mouse Hepatocytes That Release Danger Signals to Stimulate Immune Cells. Hepatology (2011) 54:133–44. doi: 10.1002/hep.24341 PMC4158408 21488066

[38] Petrasek J Bala S Csak T Lippai D Kodys K Menashy V. IL-1 Receptor Antagonist Ameliorates Inflammasome-Dependent Alcoholic Steatohepatitis in Mice. J Clin Invest (2012) 122:3476–89. doi: 10.1172/JCI60777 PMC3461900 22945633

[39] Boaru SG Borkham-Kamphorst E Tihaa L Haas U Weiskirchen R. Expression Analysis of Inflammasomes in Experimental Models of Inflammatory and Fibrotic Liver Disease. J Inflammation (Lond) (2012) 9:49. doi: 10.1186/1476-9255-9-49 PMC3599703 23192004

[40] Zhong W Rao Z Rao J Han G Wang P Jiang T. Aging Aggravated Liver Ischemia and Reperfusion Injury by Promoting STING-Mediated NLRP3 Activation in Macrophages. Aging Cell (2020) 19:e13186. doi: 10.1111/acel.13186 32666684 PMC7431827

[41] Shi C Wang Q Rao Z Shi Y Wei S Wang H. Diabetes Induces Hepatocyte Pyroptosis by Promoting Oxidative Stress-Mediated NLRP3 Inflammasome Activation During Liver Ischaemia and Reperfusion Injury. Ann Transl Med (2020) 8:739. doi: 10.21037/atm-20-1839 32647664 PMC7333130

[42] Zhang L Liu H Jia L Lyu J Sun Y Yu H. Exosomes Mediate Hippocampal and Cortical Neuronal Injury Induced by Hepatic Ischemia-Reperfusion Injury Through Activating Pyroptosis in Rats. Oxid Med Cell Longev (2019) 2019:3753485. doi: 10.1155/2019/3753485 31814872 PMC6878784

[43] Yue S Zhu J Zhang M Li C Zhou X Zhou M. The Myeloid Heat Shock Transcription Factor 1/Beta-Catenin Axis Regulates NLR Family, Pyrin Domain-Containing 3 Inflammasome Activation in Mouse Liver Ischemia/Reperfusion Injury. Hepatology (2016) 64:1683–98. doi: 10.1002/hep.28739 PMC5074868 27474884

[44] Wu Y Qiu G Zhang H Zhu L Cheng G Wang Y. Dexmedetomidine Alleviates Hepatic Ischaemia-Reperfusion Injury *via* the PI3K/AKT/Nrf2-NLRP3 Pathway. J Cell Mol Med (2021) 25:9983–94. doi: 10.1111/jcmm.16871 PMC8572787 34664412

[45] Yoshihara E. TXNIP/TBP-2: A Master Regulator for Glucose Homeostasis. Antioxid (Basel) (2020) 9:765. doi: 10.3390/antiox9080765 PMC7464905 32824669

[46] He W Ye S Zeng C Xue S Hu X Zhang X. Hypothermic Oxygenated Perfusion (HOPE) Attenuates Ischemia/Reperfusion Injury in the Liver Through Inhibition of the TXNIP/NLRP3 Inflammasome Pathway in a Rat Model of Donation After Cardiac Death. FASEB J (2018) 32:6212–27. doi: 10.1096/fj.201800028RR 29870680

[47] Xue R Qiu J Wei S Liu M Wang Q Wang P. Lycopene Alleviates Hepatic Ischemia Reperfusion Injury *via* the Nrf2/HO-1 Pathway Mediated NLRP3 Inflammasome Inhibition in Kupffer Cells. Ann Transl Med (2021) 9:631. doi: 10.21037/atm-20-7084 33987329 PMC8106004

[48] Wang Z Wang H Chen X Han S Zhu Y Wang H. Inhibiting ATP6V0D2 Aggravates Liver Ischemia-Reperfusion Injury by Promoting NLRP3 Activation *via* Impairing Autophagic Flux Independent of Notch1/Hes1. J Immunol Res (2021) 2021:6670495. doi: 10.1155/2021/6670495 33860063 PMC8024071

[49] Wang Z Han S Chen X Li X Xia N Pu L. Eva1a Inhibits NLRP3 Activation to Reduce Liver Ischemia-Reperfusion Injury *via* Inducing Autophagy in Kupffer Cells. Mol Immunol (2021) 132:82–92. doi: 10.1016/j.molimm.2021.01.028 33556710

[50] Zhang T Huang W Ma Y. Down-Regulation of TRPM2 Attenuates Hepatic Ischemia/Reperfusion Injury Through Activation of Autophagy and Inhibition of NLRP3 Inflammasome Pathway. Int Immunopharmacol (2022) 104:108443. doi: 10.1016/j.intimp.2021.108443 35021129

[51] Zarpou S Mosavi H Bagheri A Malekzadeh Shafaroudi M Khonakdar-Tarsi A. NF-kappaB and NLRP3 Gene Expression Changes During Warm Hepatic Ischemia-Reperfusion in Rats With and Without Silibinin. Gastroenterol Hepatol Bed Bench (2021) 14:267–75. PMC8245836 34221267

[52] Song SH Liu F Zhao YY Sun KY Guo M Li PL. Bruton's Tyrosine Kinase Inhibitor Attenuates Warm Hepatic Ischemia/Reperfusion Injury *via* Modulation of the NLR Family Pyrin Domain Containing 3 Inflammasome. Transplant Proc (2020) 52:2947–54. doi: 10.1016/j.transproceed.2019.10.024 33131902

[53] El-Sisi AEE Sokar SS Shebl AM Mohamed DZ Abu-Risha SE. Octreotide and Melatonin Alleviate Inflammasome-Induced Pyroptosis Through Inhibition of TLR4-NF-kappaB-NLRP3 Pathway in Hepatic Ischemia/Reperfusion Injury. Toxicol Appl Pharmacol (2021) 410:115340. doi: 10.1016/j.taap.2020.115340 33264646

[54] Cao Q Luo J Xiong Y Liu Z Ye Q. 25-Hydroxycholesterol Mitigates Hepatic Ischemia Reperfusion Injury *via* Mediating Mitophagy. Int Immunopharmacol (2021) 96:107643. doi: 10.1016/j.intimp.2021.107643 33878616

[55] Cai J Zhang X Chen P Li Y Liu S Liu Q. The ER Stress Sensor Inositol-Requiring Enzyme 1alpha in Kupffer Cells Promotes Hepatic Ischemia-Reperfusion Injury. J Biol Chem (2022) 298:101532. doi: 10.1016/j.jbc.2021.101532 34953853 PMC8760522

[56] Qin Y Wang C Xu S Wu C Wang S Pan D. G Protein-Coupled Receptor 30 Activation Protects Hepatic Ischemia-Reperfusion Injury of Liver Tissue Through Inhibiting NLRP3 in the Rat Model. J Histotechnol (2021) 44:27–36. doi: 10.1080/01478885.2020.1826175 33210578

[57] Lin Y Lin L Gao L Wang S Wu B. Rev-Erbalpha Regulates Hepatic Ischemia-Reperfusion Injury in Mice. Biochem Biophys Res Commun (2020) 529:916–21. doi: 10.1016/j.bbrc.2020.06.152 32819599

[58] Li X Wu Y Zhang W Gong J Cheng Y. Pre-Conditioning With Tanshinone IIA Attenuates the Ischemia/Reperfusion Injury Caused by Liver Grafts *via* Regulation of HMGB1 in Rat Kupffer Cells. BioMed Pharmacother (2017) 89:1392–400. doi: 10.1016/j.biopha.2017.03.022 28320107

[59] Huang R Zhao Z Jiang X Li W Zhang L Wang B. Liposomal Chrysin Attenuates Hepatic Ischaemia-Reperfusion Injury: Possible Mechanism *via* Inhibiting NLRP3 Inflammasome. J Pharm Pharmacol (2022) 74:216–26. doi: 10.1093/jpp/rgab153 34791354

[60] Gendy A Elnagar MR Soubh A Al-Mokaddem A El-Haddad A El-Sayed MK. Morin Alleviates Hepatic Ischemia/Reperfusion-Induced Mischief: *In Vivo* and *in Silico* Contribution of Nrf2, TLR4, and NLRP3. BioMed Pharmacother (2021) 138:111539. doi: 10.1016/j.biopha.2021.111539 34311537

[61] Dai J Chen Q Huang W Shi K Zhang Y Li T. Liver Kinase B1 Attenuates Liver Ischemia/Reperfusion Injury *via* Inhibiting the NLRP3 Inflammasome. Acta Biochim Biophys Sin (Shanghai) (2021) 53:601–11. doi: 10.1093/abbs/gmab030 33783473

[62] Ghoneim ME Abdallah DM Shebl AM El-Abhar HS. The Interrupted Cross-Talk of Inflammatory and Oxidative Stress Trajectories Signifies the Effect of Artesunate Against Hepatic Ischemia/Reperfusion-Induced Inflammasomopathy. Toxicol Appl Pharmacol (2020) 409:115309. doi: 10.1016/j.taap.2020.115309 33130049

[63] Vargas R Videla LA. Thyroid Hormone Suppresses Ischemia-Reperfusion-Induced Liver NLRP3 Inflammasome Activation: Role of AMP-Activated Protein Kinase. Immunol Lett (2017) 184:92–7. doi: 10.1016/j.imlet.2017.01.007 28109981

[64] Pu JL Huang ZT Luo YH Mou T Li TT Li ZT. Fisetin Mitigates Hepatic Ischemia-Reperfusion Injury by Regulating GSK3beta/AMPK/NLRP3 Inflammasome Pathway. Hepatobiliary Pancreat Dis Int (2021) 20:352–60. doi: 10.1016/j.hbpd.2021.04.013 34024736

[65] Miyauchi T Uchida Y Kadono K Hirao H Kawasoe J Watanabe T. Up-Regulation of FOXO1 and Reduced Inflammation by Beta-Hydroxybutyric Acid are Essential Diet Restriction Benefits Against Liver Injury. Proc Natl Acad Sci U.S.A. (2019) 116:13533–42. doi: 10.1073/pnas.1820282116 PMC6613133 31196960

[66] Luo Y Huang Z Mou T Pu J Li T Li Z. SET8 Mitigates Hepatic Ischemia/Reperfusion Injury in Mice by Suppressing MARK4/NLRP3 Inflammasome Pathway. Life Sci (2021) 273:119286. doi: 10.1016/j.lfs.2021.119286 33662429

[67] Ferro R Adamska A Lattanzio R Mavrommati I Edling CE Arifin SA. GPR55 Signalling Promotes Proliferation of Pancreatic Cancer Cells and Tumour Growth in Mice, and its Inhibition Increases Effects of Gemcitabine. Oncogene (2018) 37:6368–82. doi: 10.1038/s41388-018-0390-1 30061636

[68] Li F Zhang L Xue H Xuan J Rong S Wang K. SIRT1 Alleviates Hepatic Ischemia-Reperfusion Injury *via* the miR-182-Mediated XBP1/NLRP3 Pathway. Mol Ther Nucleic Acids (2021) 23:1066–77. doi: 10.1016/j.omtn.2020.11.015 PMC7887305 33664991

[69] Sinha A Clatch RJ Stuck G Blumenthal SA Patel SA. Isoflurane Hepatotoxicity: A Case Report and Review of the Literature. Am J Gastroenterol (1996) 91:2406–9. 8931426

[70] Rao Z Pan X Zhang H Sun J Li J Lu T. Isoflurane Preconditioning Alleviated Murine Liver Ischemia and Reperfusion Injury by Restoring AMPK/mTOR-Mediated Autophagy. Anesth Analg (2017) 125:1355–63. doi: 10.1213/ANE.0000000000002385 28857857

[71] Lu J Wang X Feng Z Chen Y Wen D Liu Z. The Protective Effect of Isoflurane Pretreatment on Liver IRI by Suppressing Noncanonical Pyroptosis of Liver Macrophages. Int Immunopharmacol (2021) 99:107977. doi: 10.1016/j.intimp.2021.107977 34332342

